# An index-based method for evaluating seismic retrofitting techniques. Application to a reinforced concrete primary school in Huelva

**DOI:** 10.1371/journal.pone.0215120

**Published:** 2019-04-10

**Authors:** María-Victoria Requena-García-Cruz, Antonio Morales-Esteban, Percy Durand-Neyra, João M. C. Estêvão

**Affiliations:** 1 Department of Building Structures and Geotechnical Engineering, University of Seville, Seville, Spain; 2 Instituto Universitario de Arquitectura y Ciencias de la Construcción, University of Seville, Seville, Spain; 3 Department of Civil Engineering, ISE, University of Algarve, Faro, Portugal; Pablo de Olavide University, SPAIN

## Abstract

A project named PERSISTAH (*Projetos de Escolas Resilientes aos SISmos no Território do Algarve e de Huelva*, in Portuguese) is being developed. It aims to cooperatively assess and improve the seismic vulnerability of primary schools in the Algarve (Portugal) and Huelva (Spain). A large number of schools have to be analysed. In order to determine which seismic retrofitting technique is optimal, an index-based method is presented in this paper. It considers three parameters: first, the efficiency of the seismic retrofitting technique in relation to the structural improvement obtained; second, the cost of the implementation of the retrofitting technique; and third, the architectural impact. It should be mentioned that a specific measurement for each solution according to its geometry has been performed. Also, coefficients to consider the singularities of each analysis and the importance of the parameters (number of buildings, typology, available funds, etc.) in the study are considered. The most representative primary school of Huelva has been chosen to test the index-based method. The most suitable retrofitting techniques for this type of buildings have been tested. The retrofitting technique which most increased the seismic performance has been the addition of X and V bracings within the building’s bays. Furthermore, the analyses have revealed that adding the retrofitting elements in the most vulnerable direction of the building provides a high efficiency. The results have also shown that implementing techniques of lower architectural impact gives acceptable results. The analysis of the mean damage level index has shown that the building would experiment a severe damage. All the retrofitting techniques applied have reduced it, at least, up to moderate damage. Finally, it should be noted that the position of the retrofitting elements is also paramount for providing an optimal retrofitting.

## Introduction

A European research project named PERSISTAH (*Projetos de Escolas Resilientes aos SISmos no Território do Algarve e de Huelva*, in Portuguese) is under development [1]. It aims to cooperatively assess and improve the seismic vulnerability of the primary schools located in the region of the Algarve (Portugal) and Huelva (Spain). The region is characterised by large earthquakes (M_w_≥6) of long-return periods [2]. This is due to the convergence between the Eurasian and African tectonic plates, and to the proximity of the acquainted Gibraltar-Azores fault [3].

In Spain and Portugal, schools’ buildings are very vulnerable as well the Italian Schools described in [4]. This is due to the buildings’ configurations and to their low adult/child ratio. Their configuration is characterized by the presence of short columns, soft storeys at ground floors or plan irregularities. These vulnerabilities resulted in much damage in numerous RC buildings after the 2011 Lorca (Spain) earthquake (M_w_ = 5.1) and even in collapse [5]. Moreover, most of them were constructed with Reinforced Concrete (RC) frames during the seventies. Therefore, they were mainly built prior to the current seismic codes. These characteristics make the Algarve-Huelva’s schools considerably vulnerable to earthquakes. In the case of Huelva, several typologies of buildings have been identified: linear (77 buildings), compact (88), intersecting (50), juxtaposition (4), sports (10) and prism (16) [1]. They share the same characteristics regarding the structural elements and the bays’ dimensions, the number of storeys and their height as well as the distribution in plan.

Several policies and agreements have been developed to address the seismic vulnerability of schools. Generally, they highlight they key role that schools play in creating resilient communities [6]. Such is the case of the Hyogo [7] and Sendai [8] agreements. In these, it is pointed out that solutions must be provided to strengthen schools by retrofitting. Moreover, these analyses must take into account the economic, structural and environmental impact of the solutions proposed.

**Fig 1:**
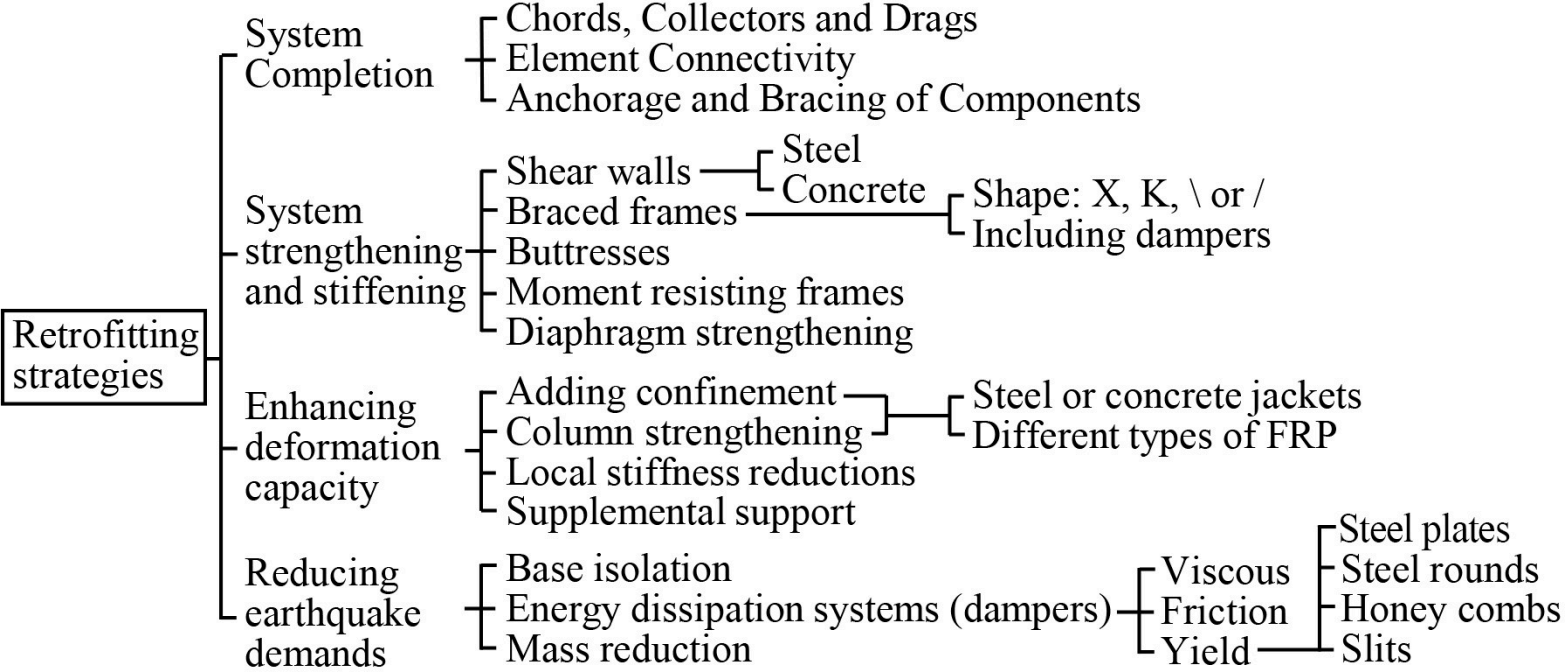
Retrofitting strategies published in the ATC-40.

There are several retrofitting techniques to improve the seismic behaviour of RC buildings. In the ATC-40 [9], a classification of these strategies was presented (Fig 1). Among them, the most implemented strategies are based on the strengthening and stiffening systems and the enhancing of the building’s deformation capacity. The reduction of the earthquake demand has also been widely studied. This is based on the addition of base-isolation devices. However, their implementation is mainly recommended in multi-storey buildings and it is very complex [10]. Therefore, in this paper, they have not been considered since the Huelva’s schools are of one to two storeys.

The strengthening and stiffening strategy is essentially based on the addition of shear walls, bracings and vertical frames within the building’s bays. The effects of adding steel bracings was experimentally tested in [11][12]. In the latter, the tests results were compared with those from nonlinear time history analyses of a building’s prototype with bracings. In [13], a comparison between the effects of implementing shear walls and bracings was carried out. It resulted in higher values of capacity for the models with bracings rather than the models with walls.

In most studies, an energy dissipation system (damper) is included within the bracings or the vertical steel frames. The effects of the different damper types have been analysed in different studies: fluid viscous in [14], friction in [15] or yield. The latter can be divided according to the dissipation element: steel plates [16], steel rounds [17], honeycombs [18] or slits [19]. The results of these analyses showed that these systems could provide a considerable improvement in the buildings’ seismic behaviour. A major part of these studies mainly performed experimental analyses.

Numerous other approaches have been proposed to enhance the buildings’ deformation capacity. In [20], the effects of the addition of RC jackets and Fibre Reinforced Polymer (FRP) wrapping in columns were assessed. Nonlinear analyses and experimental tests were carried out. The effects were compared with those derived from the addition of steel bracings and shear walls. The results showed that nonlinear static analyses could be considered as a valuable tool to assess the retrofitting interventions added to existing RC buildings. The effects of the RC jackets in columns were also experimentally compared with those from the addition of Carbon Fibre Reinforced Polymer (CFRP) in [21][22][23]. It was pointed out that the position of these measures is outstanding in order not to generate unfavourable torsional effects.

The seismic retrofitting of schools has been reported in a few studies. In [24], a new algorithm was presented to optimally obtain the amount and the position in which the FRP was needed. In [25], a fluid viscous damper bracing system was incorporated in a school. It resulted in an improvement of the seismic behaviour of up to 30%. Moreover, the authors strongly highlighted the importance of the seismic retrofitting of schools. In addition, a few studies can be found on the seismic vulnerability of schools. Most of them have been performed in Mediterranean countries i.e. Italy, Greece and Turkey. Generally, they were based on probabilistic analyses as in [26] and [27]. Also, projects focused on the performance of schools during real earthquakes have been carried out [28]. However, there is a lack of projects that aim to analyse accurately the seismic vulnerability of schools.

Regarding the construction costs, in the HAZUS [29] method, they can be considered when improving the seismic behaviour of buildings. Yet, those analyses were not as exhaustive and accurate as the analyses carried out in this work. Only standard solutions were considered in HAZUS while in this work, a specific measurement of each solution according to its geometry has been performed. Also, the construction costs have been taken into account in [30]. The authors experimentally tested a new slit damper added to RC frames.

For all the aforementioned, it can be observed that there is a lack of papers showing the efficiency of the different seismic retrofitting techniques. Moreover, despite the high amount of studies on the improvement of the seismic behaviour of RC buildings, their effects in terms of efficiency, cost and architectural impact have not been obtained and compared. Therefore, a new index-based method is proposed to obtain the most profitable solution taking into account these factors. This is especially important when evaluating a large number of buildings.

As part of the PERSISTAH research project, this paper is focused on the development of an index-based method for evaluating different seismic retrofitting techniques. It must be taken into account that there are many primary schools to evaluate in the PERSISTAH project. Therefore, this index is proposed to weigh the efficiency, the cost and the architectural impact of each retrofitting solution. In order to test the index, it is applied to a RC primary school located in Huelva. The school selected is intended to be representative of the typical primary schools located in Huelva. The effects of each retrofitting solution added to the school have been assessed. Therefore, the method will be in compliance with the requirements stablished in the Hyogo and Sendai agreements. Nonlinear static analyses have been carried out to determine the efficiency of each solution. Then, the cost has been obtained by measuring the construction costs of the solutions using a database. It should be mentioned that a specific measurement of each solution according to its geometry has been performed. Finally, each technique has been classified according to its architectural impact on the school. The results of this study allow obtaining the most profitable technique that would be the one with the highest score. The main novelty of this paper is that it aims to obtain a reproducible seismic retrofitting method to assess each technique in terms of efficiency, cost and architectural impact. It can be applied to assess any building and any retrofitting technique.

## Methodology

In this section, the fundamentals that support the methodology proposed in this paper are exposed. First, the seismic Retrofitting Index is presented (R_I_). Then, the parameters in which this index is based on are shown as well as the method of obtaining them. Next, the selected building’s configuration is presented. Finally, the seismic retrofitting techniques evaluated in this paper are discussed, including the modelling procedure and characteristics.

### The seismic retrofitting index

The R_I_ proposed in this work is based on the assessment of the efficiency, cost and architectural impact of any seismic retrofitting technique for any building. It is focused on the most outstanding aspects that affect the buildings. The goal is to achieve the most profitable solution. This is obtained through Eq (1) and it is based on the following parameters: the Efficiency Index (E_I_), the Cost Index (C_I_) and the Architectural impact Index (A_I_).

$$1.\;R_{I}=\alpha_{1}{\delta E}_{I}+\alpha_{2}\beta C_{I}+\alpha_{3}\gamma A_{I}$$ (1)

The δ, β and γ coefficients modify the main indexes according to the singularities of each situation. The procedure to obtain them is shown in the corresponding section of each index.

The α_1_, α_2_ and α_3_ coefficients are the importance factors and are explained in detail later.

#### The efficiency index (EI)

The E_I_ represents the ratio between the basal shear force resisted by the school with the retrofitting solution and the one resisted without retrofitting. The δ coefficient represents the ratio between the displacement of the building with the retrofitting and the original displacement without retrofitting. The values of shear force and displacements are obtained from the Performance Point (PP) of each situation. The performance point is based on the capacity-demand spectrum method [31] which provides the seismic performance of the buildings. This point is obtained through the intersection of the building’s capacity curve and its response spectrum as stabilised in the ATC-40 [9].

The capacity curves have been obtained through nonlinear static analyses in the two orthogonal directions of the building. Pushover analysis is reasonably successful for low and medium rise frames buildings [32]. Since the primary schools located in Huelva are of one to two storeys, this type of analysis is recommendable. Nevertheless, it is subjected to an adequate modelling of the structure and a careful selection of the lateral load distribution [33]. Therefore, as established in the EC-8 [34] and the FEMA-273 [35], two loads patterns have been considered, named pseudo-triangular and modal. The first pattern is based on lateral forces that are proportional to the total mass and the height product of each building storey. The latter pattern is based on lateral forces that are equivalent to the displacements of the predominant mode of vibration. The analyses have been carried out using the SAP2000 v.19 software [36].

RC elements nonlinear behaviour has been simulated by defining plastic hinges within the frames. As recommended in [32], default plastic hinges have been added according to the ASCE-41-13 [37]. The fracture of the frames has been considered brittle since the frames’ transverse reinforcement is not enough to represent a rigid joint. Similarly to [32][38], PM2M3 plastic hinges have been introduced in the columns while the M3 type has been used in the beams. PM2M3 plastic hinges consider the axial force and the biaxial moments while M3 plastic hinges take into account the bending moment [39]. They were introduced at the ends of the beams and the columns as in [40] and as recommended in the EC-8. Likewise, the rigid diaphragm effect of the slabs has been considered as in [41]. The contribution of the infill walls has not been considered as in [42]. By neglecting the contribution of the infills, conservative capacity curves and performance points have been obtained.

The response spectra have come from the EC-8 and the correspondent Spanish annex [43]. The a_gr_ (reference peak ground acceleration on type A ground) has been selected according to the Spanish update of the values established in [44]. Since the selected school (which will be described in detail later) is located in Almonte, the a_gr_ is 0.1g. The type of soil has been obtained from the Spanish seismic construction code of buildings (NCSE-02) [45], which considers for the location of the studied building type III. It corresponds to the type of soil C according to the EC-8. It should be noted that, according to the EC-8, schools are classified as important class III and their importance factor is 1.3. The importance factor multiplies the seismic action.

In this research, the criterion to stablish the best capacity curve has been according to the increase of the shear force resisted by the building with the seismic retrofitting technique. This criterion has been selected among others due to its simplicity and applicability. The displacement used to compare the increase of the capacity curve has been that of the original un-retrofitted building. Other criteria to establish the most efficient solution can be related to the reduction of the damage level or the displacement of the limit state, the decrease of the torsional effects, and the increase of the stiffness or the ductility.

#### The construction cost index (CI)

The C_I_ represents the ratio between the construction cost of the cheapest retrofitting solution and the cost of the assessed one. The costs have come from the measurement of each solution using a Spanish construction costs database [46] and the “*Arquímedes*” software [47]. It should be mentioned that a specific measurement of each solution according to its geometry has been performed. Moreover, the database considered is sensitive to the workmanship costs. The β coefficient is intended to reflect the importance of the cost considering the number of buildings to be retrofitted. In this case, a value of one has been used for the coefficient since only one school has been examined. The value of this parameter must be chosen carefully when a considerable number of buildings are planned to be retrofitted.

#### The architectural impact index (AI)

**Fig 2:**
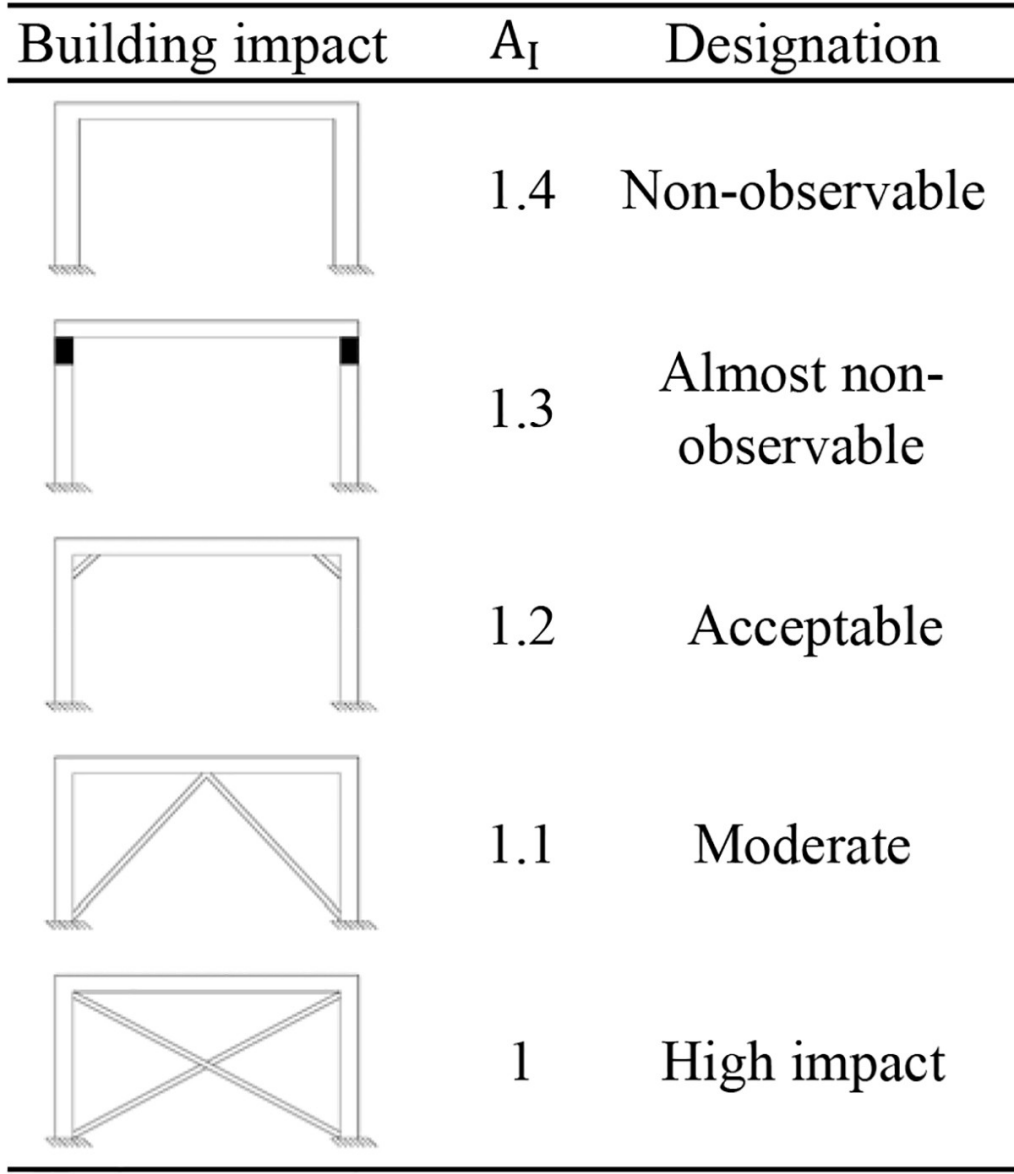
Classification of the A_I_.

The A_I_ represents the architectural impact that each retrofitting solution may have on the building. Therefore, a classification is proposed to establish the different levels of impact (Fig 2). The classification is divided into five, ranging from 1.0 for the highest architectural impact to a maximum of 1.4 for the lowest impact. The γ coefficient is related to the importance of the building in terms of protection. If the architectural impact stands out from the other two parameters (efficiency and cost), the value should be higher. That is the case of heritage buildings where the architectural impact is mandatory. In this case, the school is not protected by any standard; therefore, the selected coefficient has been one.

#### The importance factor

The α_1_, α_2_ and α_3_ coefficients are related to the importance that each index may have for each study. In this paper, they are proposed to sum 1.0. These values depend on the type of building, the available funds, the number of buildings or the repercussion that the intervention may have. Each expert must define these values according to the specific situation. In this case, the index-method has been applied to evaluate different retrofitting techniques to improve the seismic behaviour of RC schools. In this paper, owing to the seismic hazard of the area and the noticeable number of buildings, the efficiency has been considered the most important factor. Therefore, the values of α_1_, α_2_ and α_3_ coefficients are 0.6, 0.2 and 0.2, respectively.

### Building’s configuration

**Fig 3:**
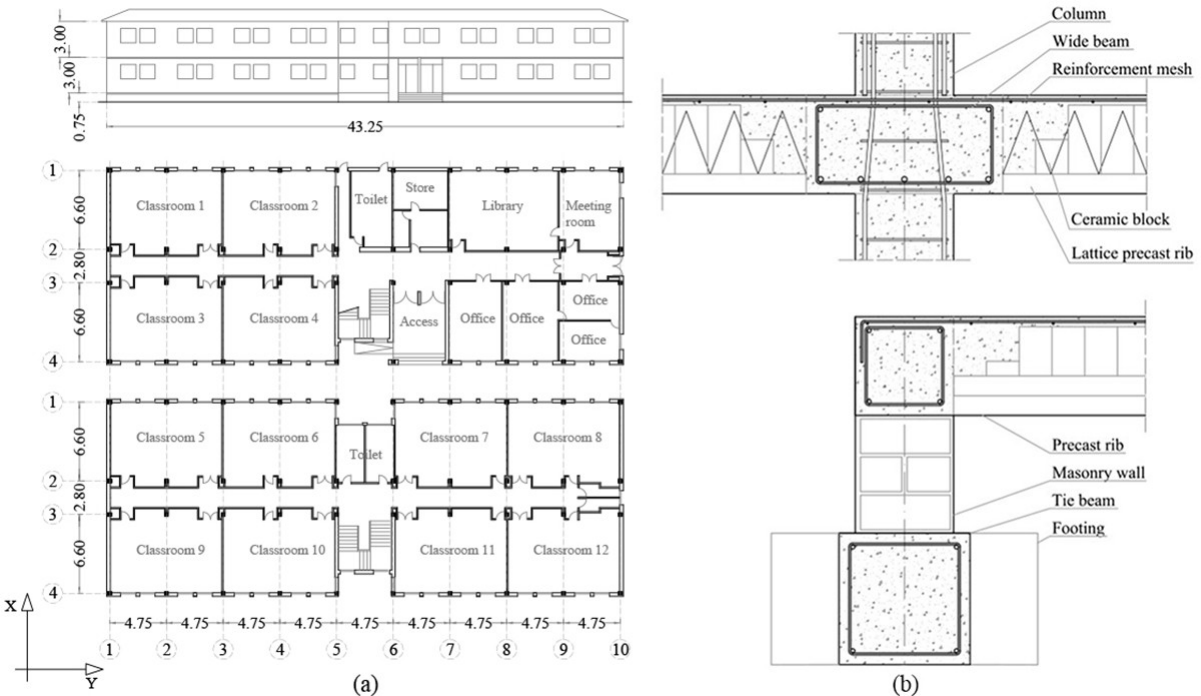
**School’s distribution in plan and façade** (dimensions in metres) **(a) and design details (b):** column-wide beam joint reinforcement detailing (superior) and insulating suspended ground floor (inferior).

**Fig 4:**
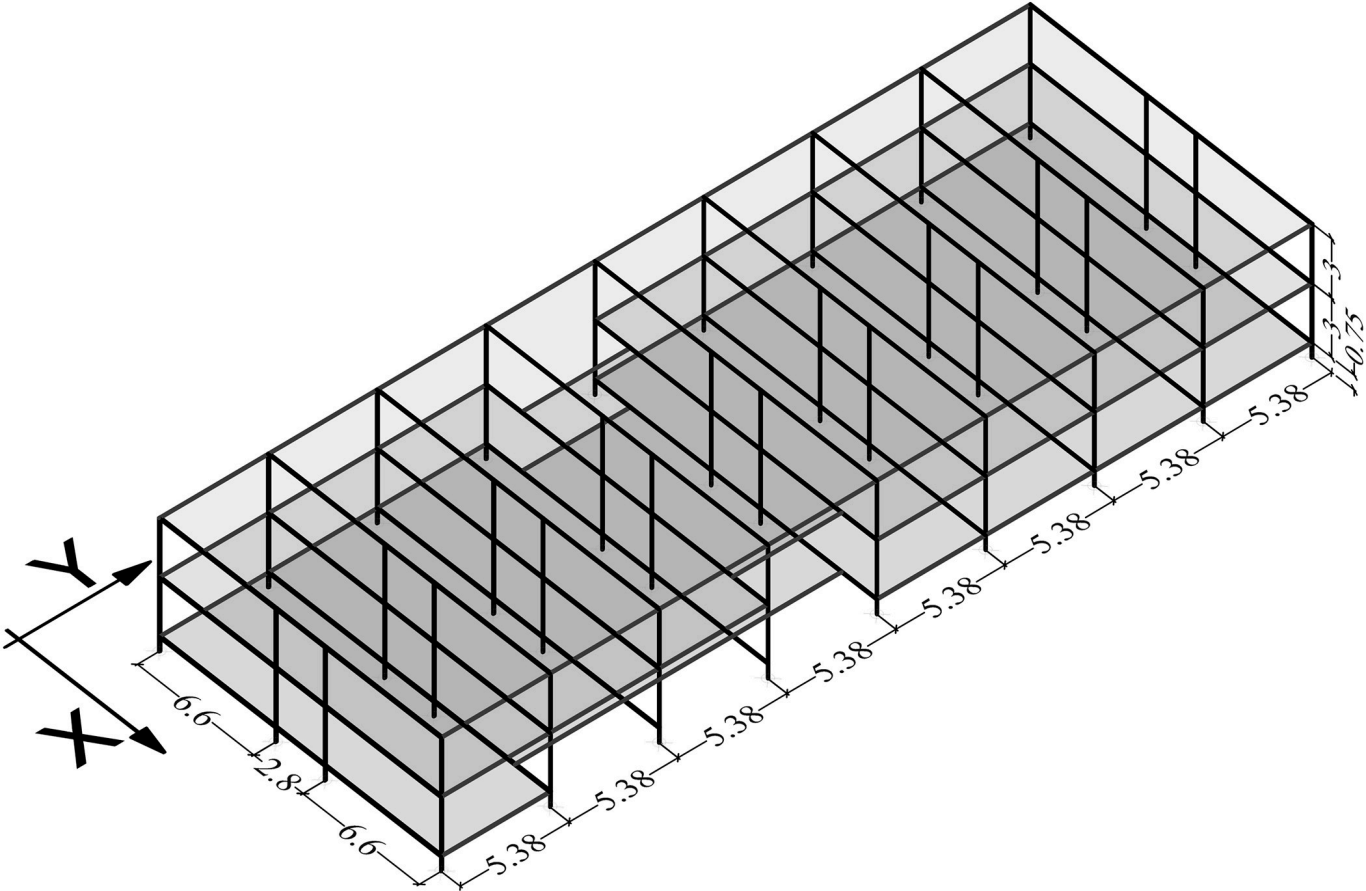
School’s configuration (dimensions in metres).

A two-storey RC frames and ribbed slabs building has been selected to be analysed (Fig 3A). It was constructed during the seventies and, therefore, designed only for gravitational loads. The school has been selected as representative of the primary schools located in Huelva. This is due to a huge amount of buildings in this area sharing the same linear typology, and similar structural and constructive characteristics i.e. 77 buildings [1]. In Fig 3B, design details of the building are provided. The RC frames characteristics are shown in Table 1. The thickness of the slabs is 30 cm and the load bearing direction of the all the ribbed slabs is the Y direction (Fig 4).

**Table 1:** Characteristics of the RC frames.

Characteristic	Columns	Load beams	Tied beams
Dimensions	30x30 cm	60x30 cm	30x30 cm
Longitudinal rebar	4Ø12 mm	Top: 2Ø12 mm	Top: 2Ø12 mm
		Lower: 5Ø16 mm	Lower: 2Ø12 mm
Transversal rebar	Ø6 mm/15 cm	Ø6 mm/20 cm	Ø6 mm/20 cm

The values of the structural materials have been obtained from the school’s original project, the Spanish technical code of buildings (CTE) [48] and the Spanish reinforced concrete code (EHE-08) [49]. The designation of the structural materials refers to the old Spanish RC codes. The RC is designated as HA-175 and the steel rebar as AEH-400. The unit weights are 24.51 kN/m^3^ and 76.47 kN/m^3^, respectively. The modulus of elasticity (E_c_) are 25,000 MPa and 200,000 MPa, respectively. The RC compressive strength (f_ck_) is 17.5 MPa while the steel minimum yield stress (F_y_) is 420 MPa.

Gravitational loads (GL) have also been obtained from the school’s data and the CTE. They were combined according to the seismic combinations and coefficients established in the NCSE-02 [45] as shown in Eq (2).

GL=W+DL+0.3Q (2)

Where W is the weight of the structural elements -i.e., RC beams and columns- DL are the dead loads -i.e., the weight of the RC ribbed slabs (3.0 kPa), the internal partitions (1 kPa), the ceiling (0.5 kPa), the ceramic flooring (1 kPa) and the infills (10 kN/m)- and Q is the live load for public spaces (3 kPa).

### Retrofitting techniques

**Fig 5:**
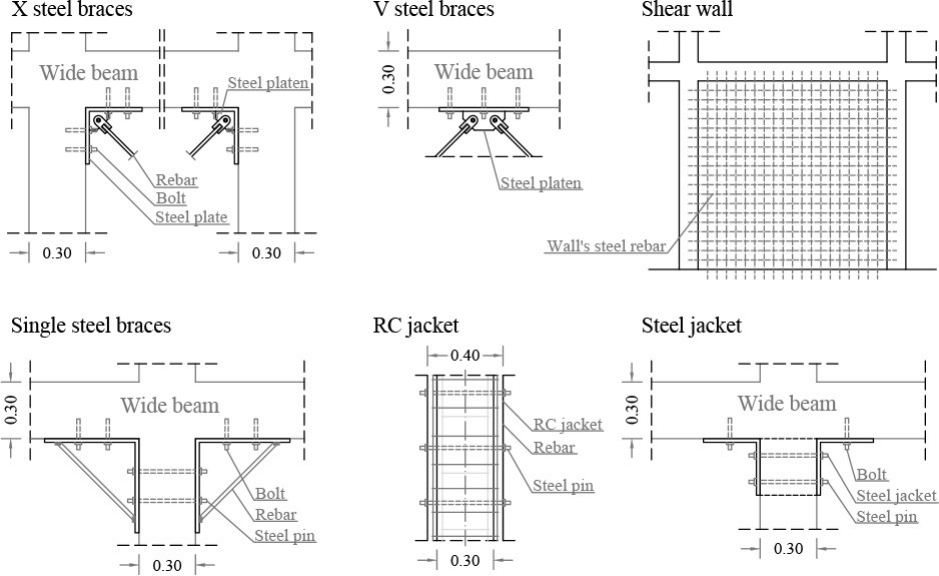
Constructive details of the retrofitting solutions proposed.

Six different retrofitting techniques have been assessed in this paper and their rehabilitation indexes have been obtained. The techniques are based on the addition of: steel braces in X and V positions, shear walls, single steel braces in the beam-column joints, and steel and RC jackets in the columns (Fig 5). These techniques have been selected since they have been widely tested in numerous studies as has been shown in the state-of-the-art Section. Notwithstanding, their efficiency has never been compared and neither have their construction costs nor their architectural impact previously been obtained. The addition of dampers has not been analysed in this work due to their higher costs.

For each technique, several models have been developed varying the position and the number of the retrofitting elements. They have been added in only one or in both buildings’ directions, in one or both storeys or in 25%, 50% or 100% of the columns in the case of the single braces and jackets. The R_I_ of each solution has been obtained and compared. The total amount of models and, therefore, R_I_ is 40: 11 models for the case of steel bracings in the X position, 8 models for the V position; 3 for the shear walls, 8 for the single steel braces, 6 for the steel jackets and 4 for the RC jackets (the designation of the models will be shown later).

The dimension of all the steel braces is Ø16 mm. The structural steel is S275. Its unit weight is 76.98 kN/m3, the E_c_ is 210,000 MPa and the F_y_ is 275 MPa. The simulation of the addition of the RC jackets has been carried out by increasing the section of the columns along their entire length and the steel rebar’s dimension by 30%. The steel jackets addition has been performed by simulating the effects of a steel plate of 30x30x0.5 cm.

The designation of the models has been determined according to the following procedure. First, the type of retrofitting element is established: steel braces in X (X) or V (V) position, shear Walls (W), Single Braces (SB), Steel (SJ) and RC Jackets (RCJ). In the case of the three first techniques, the type is followed by the number of retrofitting elements in the X or Y building’s direction as well as their position: corner (c) and middle (m). In the case of the SB and the jackets, the types’ names are followed by the percentage of columns that have been retrofitted. These are followed by the designation of the direction of the retrofitting adding X or Y after each percentage. All the names are ended with the position of the retrofitting in the storeys. These can be added only in the first floor (F1) or in both storeys (F12).

## Results

In this section, the most relevant results obtained from the models analysed are shown. First, the capacity curves for each situation are displayed. In this work, a total amount of 160 capacity curves has been obtained. The comparison percentages have been established according to the shear force of each capacity curve for the displacement of the original performance point. This is 0.06 m and 0.09 m in the X and Y direction, respectively. Then, the indexes obtained for each retrofitting technique have been established.

**Fig 6:**
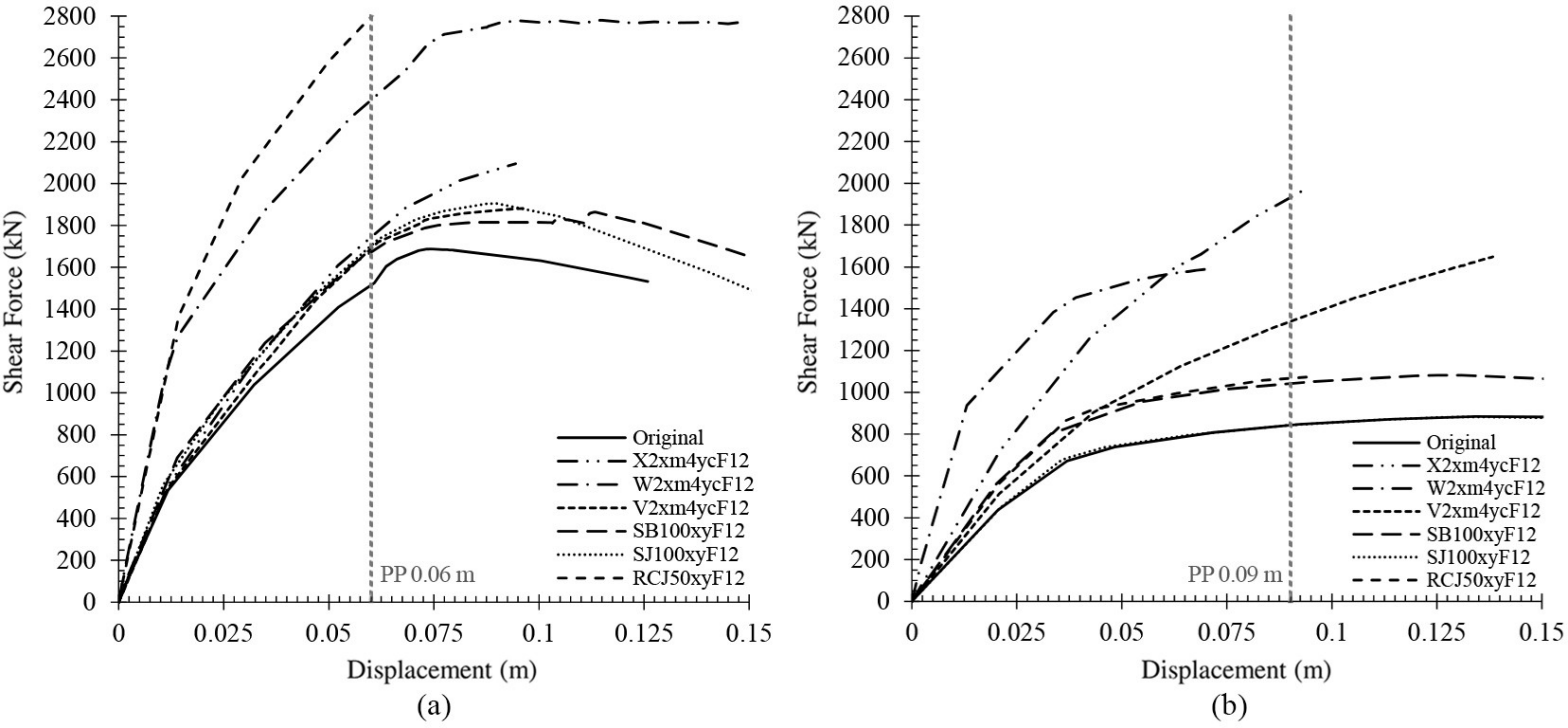
Capacity curves of the most effective solutions of each retrofitting technique in the X (a) and Y (b) directions.

The best capacity curves obtained for each retrofitting technique are shown in Fig 6. In all of them, the retrofitting elements were added in both storeys. In the case of the X direction, it can be observed that the most efficient solution was the addition of RC jackets in 50% of the columns. This improved the capacity curve by up to 75%. Nonetheless, it only improved the capacity in the Y direction by 31%. The second best solution is the addition of two shear walls in the middle of the X direction and four in the corners of the Y direction. Furthermore, this is the most efficient solution in the Y direction. Its implementation resulted in a considerable improvement of 50% and 103% in the X and Y direction, respectively. In the case of the Y direction, the second most efficient solution is adding X braces in the same position as the previous shear walls’ technique. All the same, it only improved the capacity curve in 12.5% and 110% in the X and Y direction, respectively.

**Fig 7:**
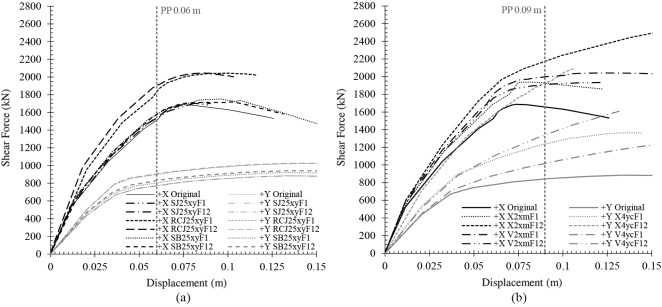
Capacity curves of the models adding techniques of minimum (a) and maximum (b) architectural impact in both directions.

In Fig 7, the capacity curves of the models that added retrofitting techniques of minimum (Fig 7A) and maximum (Fig 7B) architectural impact are plotted. First, in Fig 7A, solutions that added the retrofitting elements in the same percentage (25%) have been selected to be compared. The most efficient solutions of minimum architectural impact have been those that added RCJ and SB in both storeys. These resulted in an improvement of 15% and 9% in the X direction and of 18% and 5% in the Y direction, respectively. The rest of solutions have not generated a significant improvement.

Then, in Fig 7B, models that added a similar number of retrofitting elements of maximum architectural impact have been selected. Furthermore, the intention has been to obtain the differences between adding the bracings in only one or in the two storeys. The most efficient solution has been the addition of two X braces in the middle of the X direction and in both storeys. An improvement of 18% has been obtained in this direction. In the case of the Y direction, the most efficient solution has been the addition of four X braces in the corner and in both storeys, resulting in an improvement of 97%.

In Tables 2, 3 and 4, the efficiency, the construction costs and the architectural impact of each model are listed, respectively. The mean damage level index (DI) has been calculated according to the EC-8 and to [50] (Table 2). This classification states: (DS0) no damage, (DS1) slight damage, (DS2) moderate damage, (DS3) severe damage and (DS4) collapse. The DI of the real building is 3.03 which corresponds to the Damage State 3 (DS3), severe damage.

**Table 2:** The efficiency of each model.

Solution	α1	δx	E_I_x	δy	E_I_y	α1*δ*E_I_	DI	Solution	α1	δx	E_I_x	δy	E_I_y	α1*δ*E_I_	DI
X2xm4ycF12	0.60	0.932	1.168	0.761	1.948	0.771	1.87	SB25xyF12	0.60	0.955	1.042	0.994	1.053	0.613	2.72
X4ycF12	0.60	1.008	0.971	0.783	1.985	0.760	2.43	SB25yF12	0.60	0.977	1.010	0.994	1.058	0.612	2.71
V2xm4ycF12	0.60	0.962	1.114	0.939	1.515	0.748	2.45	V2xmF12	0.60	0.955	1.092	0.983	1.007	0.610	2.46
X2xc2ycF12	0.60	0.924	1.152	0.844	1.524	0.705	2.10	SB25yF1	0.60	0.992	0.997	1.011	1.030	0.609	2.78
V4ycF12	0.60	1.000	0.977	0.906	1.489	0.698	2.60	SB25xyF1	0.60	0.970	1.025	0.983	1.028	0.601	2.78
X2xm4ycF1	0.60	0.962	1.052	0.911	1.415	0.690	2.74	X2xcF1	0.60	0.939	1.074	0.989	1.007	0.601	2.61
X2ymF12	0.60	1.000	0.985	0.861	1.524	0.689	2.68	SJ100xyF12	0.60	0.939	1.063	0.994	1.001	0.598	2.79
X4ycF1	0.60	1.000	0.988	0.911	1.412	0.682	2.82	SJ50xyF1	0.60	0.985	1.008	1.000	0.998	0.597	2.92
V2ymF12	0.60	1.000	0.984	0.961	1.266	0.660	2.64	SJ25xyF1	0.60	0.985	1.000	0.994	1.000	0.594	2.79
V2xm4ycF1	0.60	0.970	1.028	0.994	1.206	0.659	2.63	SB25xF12	0.60	0.955	1.040	0.983	1.001	0.593	2.83
V4ycF1	0.60	0.992	0.998	0.983	1.198	0.650	2.75	W2xm4ycF12	0.60	0.689	1.299	0.589	1.830	0.592	2.16
X2ymF1	0.60	0.977	1.005	0.944	1.247	0.648	2.66	RCJ25xyF1	0.60	0.856	1.072	0.928	1.135	0.591	2.68
RCJ50xyF12	0.60	0.682	1.461	0.900	1.238	0.633	2.34	SJ50xyF12	0.60	0.955	1.034	1.000	0.979	0.590	2.91
V2ymF1	0.60	0.985	0.990	1.011	1.118	0.631	2.67	SJ25xyF12	0.60	0.970	1.011	0.989	0.996	0.590	2.85
SB100xyF12	0.60	0.886	1.110	0.894	1.222	0.623	2.57	SB25xF1	0.60	0.970	1.006	0.983	1.003	0.589	2.78
X2xcF12	0.60	0.924	1.151	1.000	1.009	0.622	2.28	SB100xyF1	0.60	0.924	1.015	0.972	1.044	0.586	2.60
X2xmF12	0.60	0.909	1.166	1.000	1.011	0.621	2.41	SJ100xyF1	0.60	0.970	1.005	0.983	0.994	0.586	2.77
V2xmF1	0.60	0.955	1.090	1.006	1.013	0.618	2.55	RCJ25xyF12	0.60	0.795	1.104	0.933	1.140	0.583	2.66
W4ycF12	0.60	0.939	1.062	0.583	1.799	0.614	2.25	RCJ50xyF1	0.60	0.765	1.184	0.839	1.228	0.581	2.40
X2xmF1	0.60	0.955	1.070	1.011	1.012	0.613	2.65	W2xmF12	0.60	0.712	1.230	0.994	1.060	0.579	2.41

**Table 3:** The construction cost of each model.

Solution	α2	β	C_I_	α2*β*C_I_	Solution	α2	β	C_I_	α2*β*C_I_
SJ25xyF1	0.20	1.00	1.000	0.200	SB25xyF1	0.20	1.00	0.149	0.030
V2xmF1	0.20	1.00	0.741	0.148	V2xm4ycF1	0.20	1.00	0.144	0.029
X2xmF1	0.20	1.00	0.732	0.146	X2xm4ycF1	0.20	1.00	0.142	0.028
SJ25xyF12	0.20	1.00	0.500	0.100	RCJ25xyF12	0.20	1.00	0.121	0.024
V2xmF12	0.20	1.00	0.371	0.074	SJ100xyF1	0.20	1.00	0.118	0.024
SB25yF1	0.20	1.00	0.370	0.074	SB25xF12	0.20	1.00	0.115	0.023
X2xmF12	0.20	1.00	0.366	0.073	V4ycF12	0.20	1.00	0.089	0.018
V2ymF1	0.20	1.00	0.357	0.071	X4ycF12	0.20	1.00	0.088	0.018
X2ymF1	0.20	1.00	0.351	0.070	X2xc2ycF12	0.20	1.00	0.088	0.018
X2xcF1	0.20	1.00	0.351	0.070	SJ50xyF12	0.20	1.00	0.082	0.016
SB25xF1	0.20	1.00	0.244	0.049	SB25xyF12	0.20	1.00	0.074	0.015
RCJ25xyF1	0.20	1.00	0.242	0.048	SB100xyF1	0.20	1.00	0.072	0.014
W2xmF12	0.20	1.00	0.205	0.041	V2xm4ycF12	0.20	1.00	0.072	0.014
SB25yF12	0.20	1.00	0.185	0.037	X2xm4ycF12	0.20	1.00	0.071	0.014
V4ycF1	0.20	1.00	0.179	0.036	RCJ50xyF1	0.20	1.00	0.061	0.012
V2ymF12	0.20	1.00	0.179	0.036	SJ100xyF12	0.20	1.00	0.059	0.012
X4ycF1	0.20	1.00	0.176	0.035	W4ycF12	0.20	1.00	0.053	0.011
X2ymF12	0.20	1.00	0.176	0.035	W2xm4ycF12	0.20	1.00	0.042	0.008
X2xcF12	0.20	1.00	0.176	0.035	SB100xyF12	0.20	1.00	0.036	0.007
SJ50xyF1	0.20	1.00	0.164	0.033	RCJ50xyF12	0.20	1.00	0.030	0.006

**Table 4:** The architectural impact of each model.

Solution	α3	γ	A_I_	α3*γ*A_I_	Solution	α3	γ	A_I_	α3*γ*A_I_
SJ25xyF1	0.20	1.00	1.400	0.280	SB100xyF12	0.20	1.00	1.225	0.245
RCJ25xyF1	0.20	1.00	1.400	0.280	X4ycF1	0.20	1.00	1.200	0.240
SJ25xyF12	0.20	1.00	1.375	0.275	X2ymF1	0.20	1.00	1.200	0.240
RCJ25xyF12	0.20	1.00	1.375	0.275	X2xmF1	0.20	1.00	1.200	0.240
SB25yF1	0.20	1.00	1.350	0.270	X2xcF1	0.20	1.00	1.200	0.240
SB25xyF1	0.20	1.00	1.350	0.270	V4ycF12	0.20	1.00	1.200	0.240
SB25xF1	0.20	1.00	1.350	0.270	V2ymF12	0.20	1.00	1.200	0.240
SJ50xyF1	0.20	1.00	1.350	0.270	V2xmF12	0.20	1.00	1.200	0.240
RCJ50xyF1	0.20	1.00	1.350	0.270	X4ycF12	0.20	1.00	1.150	0.230
SB25yF12	0.20	1.00	1.325	0.265	X2ymF12	0.20	1.00	1.150	0.230
SB25xyF12	0.20	1.00	1.325	0.265	X2xmF12	0.20	1.00	1.150	0.230
SB25xF12	0.20	1.00	1.325	0.265	X2xcF12	0.20	1.00	1.150	0.230
SJ50xyF12	0.20	1.00	1.325	0.265	V2xm4ycF1	0.20	1.00	1.150	0.230
RCJ50xyF12	0.20	1.00	1.325	0.265	W4ycF12	0.20	1.00	1.150	0.230
SJ100xyF1	0.20	1.00	1.300	0.260	W2xmF12	0.20	1.00	1.150	0.230
SJ100xyF12	0.20	1.00	1.275	0.255	X2xm4ycF1	0.20	1.00	1.100	0.220
V4ycF1	0.20	1.00	1.250	0.250	V2xm4ycF12	0.20	1.00	1.100	0.220
V2ymF1	0.20	1.00	1.250	0.250	W2xm4ycF12	0.20	1.00	1.100	0.220
V2xmF1	0.20	1.00	1.250	0.250	X2xm4ycF12	0.20	1.00	1.050	0.210
SB100xyF1	0.20	1.00	1.250	0.250	X2xc2ycF12	0.20	1.00	1.050	0.210

The most efficient solution has been the addition of two X braces in the middle of the X direction and four in the corner of the Y direction in both storeys. By contrast, the least efficient solution has been including two walls in the middle of the X direction in both storeys. Conversely, the cheapest solution has been the addition of SB in 25% of the columns of one storey. Moreover, it has been also the solution of minimum architectural impact and higher R_I_ (Table 5). The most expensive solution has been the inclusion of RCJ in 50% of the columns in both storeys. The solution of worst architectural impact has been the addition of two X braces in the corner of both directions and storeys. Finally, the solution of lowest R_I_ has been adding two walls in the middle of the X direction and four in the corner of the Y direction in both storeys.

**Table 5:** Rehabilitation index of each model.

Solution	R_I_	Solution	R_I_
SJ25xyF1	1.074	V2xm4ycF1	0.917
V2xmF1	1.016	SB25yF12	0.914
X4ycF12	1.008	X2xcF1	0.912
X2xmF1	1.000	SB25xF1	0.907
X2xm4ycF12	0.996	RCJ50xyF12	0.904
V2xm4ycF12	0.983	SB25xyF1	0.901
SJ25xyF12	0.965	SJ50xyF1	0.900
X2ymF1	0.958	SB25xyF12	0.893
X4ycF1	0.958	X2xcF12	0.887
V4ycF12	0.955	RCJ25xyF12	0.882
X2ymF12	0.954	SB25xF12	0.881
SB25yF1	0.953	SB100xyF12	0.875
V2ymF1	0.953	SJ50xyF12	0.871
X2xm4ycF1	0.939	SJ100xyF1	0.870
V4ycF1	0.936	SJ100xyF12	0.865
V2ymF12	0.936	RCJ50xyF1	0.863
X2xc2ycF12	0.933	W4ycF12	0.855
X2xmF12	0.924	SB100xyF1	0.850
V2xmF12	0.924	W2xmF12	0.850
RCJ25xyF1	0.920	W2xm4ycF12	0.820

## Analysis of the results

In this section, the results from the pushover analyses are analysed and compared. Then, the values obtained for the efficiency, cost and architectural impact obtained for each solution are analysed as well as the results obtained for the R_I_.

The results from the pushover analyses have revealed several differences between the capacity curves of the models including the same and different retrofitting techniques. The addition of both X and V bracings in one direction barely improved the capacity curve in the other direction. A similar result has been obtained adding jackets, despite being implemented in both directions. However, adding shear walls in only one direction noticeably improved the capacity curve in the other direction up to 30%. This may be due to the considerable thickness of the walls in the other direction. The addition of SJ in the X direction resulted in a noticeable improvement while adding RCJ has been proved considerably efficient in this direction, attaining up to 75%. In both cases, the behaviour in the Y direction did not improve.

Conversely, several differences can also be observed from implementing the retrofitting elements in one or in both storeys. This has been outstanding in the case of the X and the V bracings. A maximum 40% of improvement resulted from comparing the effects of adding the elements in only one and in both storeys. Yet, it has not been noticeable when adding jackets in one or both storeys. As for the percentage of implementation in columns, these results revealed that the addition of SB led to capacity curves of acceptable improvement of the capacity curve in the Y direction. Nevertheless, adding SJ and RCJ has been more efficient.

Regarding the efficiency, it can be observed that the most efficient techniques are mainly the addition of steel bracings in both X and V positions. Notwithstanding, the latter resulted in lower values of capacity curves and, therefore, efficiency. The number and position of the retrofitting elements have been determining to improve the efficiency. Since the worst direction of the building has been the Y, the solutions that only added elements in this direction have had higher values rather than those that included fewer elements in both directions. The next effective solution has been adding SB in a considerable percentage, the addition of jackets being the least efficient technique.

In the case of cost, the cheapest techniques have been the implementation of SJ and SB in columns. Nonetheless, adding V bracings in one or in both storeys and in only one direction had an acceptable cost, resulting in better results than adding X bracings. Moreover, considerable differences can be found when adding the elements in one or in both storeys for the same configuration.

The solutions of higher A_I_ have been the addition of jackets and SB. The solutions of higher impact have been those based on the addition of X and V bracings and walls. All the same, the V bracings have produced lower values of impact than the X bracings.

The aforementioned notes that the solution of higher R_I_ has been the SJ25xyF1. This added SB in 25% of the columns of one storey. It has been the most profitable solution due to its minimum cost and architectural impact, despite not being the most effective solution. It is followed by models that implemented V and X bracings mainly in the Y direction. This has been due to the considerable improvement of the capacity curves in that direction. Adding V bracings led to better results of R_I_ since their cost and architectural impact are lower than the values for the X bracings. Solutions that added SB in less than 50% of the columns obtained acceptable values of R_I_. Adding jackets has not been proved to be a profitable solution since their efficiency has not been considerable enough to compensate their high costs. In order to obtain considerable values of efficiency, they had to be added in a high percentage of columns, therefore, increasing their costs.

## Conclusions

The region of Huelva is relevant due to its seismic hazard. A study of the area’s school buildings has revealed that they have one of the most seismically vulnerable building typologies. Therefore, a solution is necessary to improve their seismic behaviour in case of an earthquake. In this paper, a new index-based method for assessing different seismic retrofitting techniques has been presented and applied to a representative school in Huelva. This is based on the efficiency, cost and architectural impact of each solution in order to comply with the Hyogo and Sendai agreements. It can be reproduced to assess and compare any building’s typology and any retrofitting technique. The results have shown that this method is robust and has successfully achieved the goal proposed.

The nonlinear static analyses have revealed that just adding retrofitting elements in the most vulnerable direction of the building can lead to higher values of efficiency than including fewer elements but in both directions. The addition of bracings, jackets and single braces in only one direction did not improve the behaviour of the other direction. Contrariwise, the implementation of walls produced improvements in both directions.

The R_I_ values obtained for each solution have been compared and this has resulted in the most profitable solutions having been the addition of both X and V bracings. Adding single steel braces has also been proved to be an acceptable technique to be implemented in the retrofitting process of buildings. Moreover, it is concluded that the number and position of the retrofitting elements have been determinant in obtaining higher R_I_ values. It is also noticeable that adding steel and RC jackets have been the least profitable techniques due to their low values of efficiency and high costs.

It has also been demonstrated that it is not necessary to add the retrofitting elements in every column or bay of the building. Selecting the most effective positions for the retrofitting element implementation should be carefully carried out to obtain a profitable improvement.

The analysis of the DI, according the EC-8 and to [50], has shown that the building would experiment a severe damage (DS3). All the retrofitting techniques applied have reduced it up to moderate damage (DS2). Moreover, the solution X2xm4ycF12 has reduced it up to DS1 (slight damage), showing a remarkable improvement regarding the DI.

Finally, the authors want to point out that further research could be carried out to determine the E_I_ considering other factors such as the damage level, the displacement of the limit state, the torsional effects, the stiffness and the ductility.
